# Characterizing Sjögren-Associated Fatigue: A Distinct Phenotype from ME/CFS

**DOI:** 10.3390/jcm12154994

**Published:** 2023-07-29

**Authors:** Laura Kim, Claudia Kedor, Frank Buttgereit, Harald Heidecke, Desiree Schaumburg, Carmen Scheibenbogen

**Affiliations:** 1 Institute of Medical Immunology, Charité—Universitätsmedizin Berlin, 13353 Berlin, Germany; claudia.kedor@charite.de (C.K.); carmen.scheibenbogen@charite.de (C.S.); 2 Department of Rheumatology and Clinical Immunology, Charité—Universitätsmedizin Berlin, 10117 Berlin, Germany; frank.buttgereit@charite.de (F.B.); desiree.schaumburg@gmail.com (D.S.); 3 CellTrend GmbH, Im Biotechnologiepark 3, 14943 Luckenwalde, Germany; heidecke@celltrend.de

**Keywords:** fatigue, autoantibodies, Sjogren′s Syndrome, myalgic encephalomyelitis, chronic fatigue syndrome, hand grip strength, post exertional malaise

## Abstract

Fatigue is the most commonly reported and debilitating extraglandular symptom of primary Sjögren′s syndrome (pSS). Fatigue and exertional intolerance are hallmark symptoms of myalgic encephalomyelitis/chronic fatigue syndrome (ME/CFS). We aimed to characterize fatigue and further symptoms among pSS patients and to determine whether there is a symptom overlap in pSS and ME/CFS. In 19 patients with pSS, we assessed pSS symptom severity and disease activity via questionnaires as well as the Canadian Consensus Criteria (CCC) for ME/CFS. Hand grip strength (HGS) and levels of α1-, α2-, β1-, β2-, M3- and M4-receptor-autoantibodies were measured. A subgroup of pSS patients exhibited severe fatigue and had higher severity of pain (*p* = 0.045), depression (*p* = 0.021) and sleep disturbances (*p* = 0.020) compared to those with less fatigue. Four of eighteen pSS patients fulfilled the CCC. HGS parameters strongly correlated with fatigue severity (*p* < 0.05), but strength fully recovered one hour after exertion in contrast to ME/CFS. Levels of β1-, β2- and M4-receptor-autoantibodies were elevated and correlated significantly with disease activity assessed by the ESSDAI (*p* < 0.05), but not fatigue severity. Only a minor subgroup of pSS patients fulfills the CCC, and post exertional malaise (PEM) is atypical, as it is primarily triggered by mental/emotional but not physical exertion. HGS assessment is an objective measure to assess overall fatigue severity.

## 1. Introduction

Primary Sjögren′s Syndrome (pSS) is a chronic inflammatory autoimmune disease with an estimated prevalence of 61 per 100,000 [1]. Up to 98% of patients with pSS exhibit sicca symptoms, which are the most common manifestation of the disease due to its predisposition to affect the exocrine glands [2]. The pSS may additionally cause numerous extraglandular manifestations [3]. Fatigue is present in as many as two-thirds of pSS patients [4], who describe it as the most debilitating feature of their disease [5]. A subgroup of myalgic encephalomyelitis/chronic fatigue syndrome (ME/CFS) patients is known to display sicca symptoms, the cardinal symptom of pSS, and even fulfill diagnosis criteria for seronegative pSS [6,7]. This led to the speculation that the two disorders share common pathophysiological and clinical features.

ME/CFS is an acquired multi-system disease that causes significant functional impairment and drastically impacts patients′ quality of life [8]. The prevalence was estimated at 0.2–0.8% prepandemic [9]. Cardinal symptoms of ME/CFS include fatigue and exertional intolerance with worsening of symptoms after physical, cognitive or emotional exertion, referred to as ‘post exertional malaise’ (PEM) [10]. Additionally, ME/CFS is characterized by the presence of pain, sleep disorders, neurological and cognitive impairment as well as autonomic and immunological dysfunction [10]. Notably, these symptoms of ME/CFS are also highly prevalent in pSS [11,12]

This overlap in clinical presentation of pSS and ME/CFS may pose a diagnostic challenge. Therefore, our study aimed to provide a deeper characterization of the clinical presentation of pSS, specifically in terms of fatigue and exertional intolerance.

In the past, fatigue has been evaluated solely using subjective questionnaires, but recently, there has been an increased effort to objectively quantify fatigue severity. In patients with ME/CFS, as well as patients with tumor related fatigue, repeated hand grip strength (HGS) assessment has been used as a sensitive diagnostic test to assess muscular fatigue and fatigability [13]. Reduction in HGS has also been shown to be associated with fatigue in patients with other chronic conditions, such as primary biliary cirrhosis [14] and chronic obstructive pulmonary disease [15]. Additionally, higher PEM in ME/CFS correlated with lower HGS and reduced recovery of HGS [13]. We therefore included HGS assessment as an objective marker of physical fatigue.

Both pSS and ME/CFS are triggered by infections in most patients [16,17]. In pSS, there is strong evidence for an autoimmune mechanism with antibodies to the autoantigens Ro/SSA and La/SSB, among others, and an increased activation of both the innate and adaptive immune system, with B cells playing an important role in autoantibody production and formation of ectopic germinal center-like structures [18]. Although it remains a subject of debate whether ME/CFS is an autoantibody-mediated disease [19], it is accompanied by a dysregulation of immune system homeostasis. This includes the decreased function of natural killer (NK) cells, an altered cytokine profile and the correlation of autoantibodies [20] directed against neurotransmitter receptors with immune alterations and symptom severity [21,22]. In particular, elevated ß1 and ß2 adrenergic receptor autoantibodies (ß1/ß2-AdR-AAB) and M3/M4 acetylcholine receptor autoantibodies (M3R/M4R-AAB) have been found in subsets of ME/CFS patients [21,23]. These are thought to impact autonomic nervous system function and vasoregulation [24]. Additionally, ß1/ß2-AdR-AAB blood levels correlated with structural alterations in the brain that were related to pain modulation [25]. When removing IgG from circulation via immunoadsorption, short-term clinical improvement was observed in most patients [26,27].

Dysfunction of the autonomous nervous system is a common feature of both ME/CFS and pSS that is associated with disease activity and fatigue severity [28,29]. Additionally, the presence of M3R-AchR-AAB has already been reported in 60–80% of pSS patients. However, whether they play a role in the pathogenesis of pSS and whether they influence autonomic nervous system function remains elusive [30]. There is a lack of extensive research on the presence of other antibodies, such as ß1/ß2-AdR-AAB, in pSS. Therefore, we also investigated the presence of and correlation with disease and symptom severity of these autoantibodies in pSS patients.

## 2. Materials and Methods

### 2.1. Patients

A total of 19 patients diagnosed with pSS, who presented at the outpatient clinic for rheumatology at the Department of Rheumatology and Clinical Immunology at the Charité Universitätsmedizin Berlin between February 2020 and March 2021, were included in this study as part of the Rh-GIOP (Glucocorticoid-Induced Osteoporosis in Patients With Chronic Inflammatory Rheumatic Diseases) project. The project is an ongoing prospective cohort study started in 2015. It was approved by the ethical committee of the Charité Universitätsmedizin Berlin (EA1/367/14). All patients gave informed consent to participate in this study before taking part. Diagnosis of pSS in all patients was based on the 2016 ACR-EULAR Classification Criteria for primary Sjögren’s Syndrome [31]. The ACR-EULAR Classification Criteria for primary Sjögren′s syndrome require individuals obtain a score of four or higher based on weighted items, including labial salivary gland inflammation, presence of anti-SSA antibodies, ocular staining score, Schirmer test results and saliva flow rate while not meeting any of the exclusion criteria. Further inclusion criteria were (1) at least 18 years of age, (2) current or previous treatment with gluococorticoids and (3) eligibility for Osteoporosis diagnostics as recommended by the Dachverband Osteologie [32], whereas key exclusion criteria were (1) pregnancy or lactation, (2) inability to provide informed consent for any reason, (3) inability to perform HGS assessment for any reason and (4) current other acute inflammatory or rheumatic disease apart from pSS.

### 2.2. Questionnaires for Symptom Scoring

Indices of pSS symptom severity (ESSPRI) and disease activity (ESSDAI) were calculated for all patients [33]. The presence and severity of symptoms of ME/CFS in patients with pSS were assessed based on the 2003 Canadian Consensus Criteria (CCC) [10]. In addition, severity of perceived fatigue was quantified by a numeric rating scale (NRS) from 0 to 10 and the Multidimensional Fatigue Inventory (MFI) [34,35]. The severity of other symptoms, such as anxiety and depression, sleep quality and physical activity, were evaluated using the following questionnaires: Hospital Anxiety and Depression Scale (HADS) [36], Pittsburgh Sleep Quality Index (PSQI) [37] and International Physical Activity Questionnaire (IPAQ-SF) [38]. Symptoms of autonomic dysfunction were assessed by the Composite Autonomic Symptom Score 31 (COMPASS 31) [39].

### 2.3. Hand Grip Strength Measurement

HGS of the dominant hand was measured using a digital hand dynamometer (Deyard ^®^, model: EH101) in two separate sessions. Rest time between sessions was exactly 60 min, in which no strenuous physical activity took place. Patients sat in an upright position facing a standard table during measurements of HGS. The forearm of the dominant hand was placed on the table in full supination holding the dynamometer. Under supervision and verbal motivation, the handle was pulled 10 times with maximum force for three seconds, followed by a five-second relaxation phase. Before starting the measurement, the participants were shown two separate demonstrations of how the dynamometer should be used. The dynamometer displays the highest value reached within these three seconds (measurement in kg), and this single value is then recorded. The attempt with the highest reading out of ten repetitions was recorded as the maximum strength (Fmax). Mean force (Fmean), Fatigue Ratio (Fmax/Fmean) and Recovery Ratio (Fmean2/Fmean1) were also calculated [13].

### 2.4. Determination of Autoantibody Levels and Laboratory Blood Data

CellTrend GmbH, Luckenwalde, Germany, analyzed serum levels of autoantibodies against α1-, α2-, β1-, β2-adrenergic receptors (AdR) and M3- and M4-acetylcholinergic receptors (AchR). Whole blood samples from each subject were allowed to clot at room temperature and then centrifuged at 2000× *g* for 15 min in a refrigerated centrifuge. The serum was purified and stored at −35 °C. The AAB were measured in serum samples using a sandwich ELISA kit (CellTrend GmbH, Luckenwalde, Germany). The microtiter 96-well polystyrene plates were coated with full-length receptor proteins. To maintain the conformational epitopes of the receptor, 1 mM calcium chloride was added to every buffer. Duplicate samples of a 1:100 serum dilution were incubated at 4 °C for 2 h. After washing steps, plates were incubated for 60 min with a 1:20,000 dilution of horseradish-peroxidase labelled goat anti-human IgG used for detection. In order to obtain a standard curve, the plates were incubated with test serum from a GPCR autoantibody-positive index patient. The ELISAs were validated according to the FDA′s “Guidance for industry: Bioanalytical method validation”. The upper normal values were defined based on validation studies in healthy controls.

Blood tests necessary for calculating the ESSDAI were determined at Charité diagnostics laboratory Labor Berlin GmbH.

### 2.5. Statistical Analysis

Statistical data analyses were performed using IBM SPSS Statistics 27.0 (New York, NY, USA and R 4.2.1 (R Foundation for Statistical Computing, Vienna, Austria, http://www.R-project.org, accessed on 1 September 2022). All data are presented as median and interquartile range (IQR), mean and standard deviation (SD) or frequency (*n*) and percentage where appropriate. Comparisons of quantitative parameters between two groups were performed using the nonparametric Mann–Whitney U test and for HGS test series comparison using the parametric paired t-test. Correlation analysis was performed using the nonparametric Spearman coefficient. Additionally, a hierarchical cluster analysis, to group patients by fatigue severity, and a receiver operating characteristic (ROC) analysis, to test accuracy of HGS measurement, were performed. The *p*-values < 0.05 were considered to provide evidence for a statistically significant result. The *p*-values are descriptive, as there was no correction for multiple testing due to the low number of patients.

## 3. Results

### 3.1. Patient Characteristics and Symptom Presentation

We analyzed a cohort of 19 pSS patients. Patient characteristics are shown in Table 1.

Patients rated their fatigue as a median of 4 on a NRS from 0 to 10. As shown by the MFI, patients had a median of 12 points in the ‘general fatigue’ domain, the possible range being 4–20 points. Physical fatigue, portrayed by the MFI domains ‘physical fatigue’ and ‘reduced activity’, contributed more to the overall fatigue than mental fatigue.

**Figure 1:**
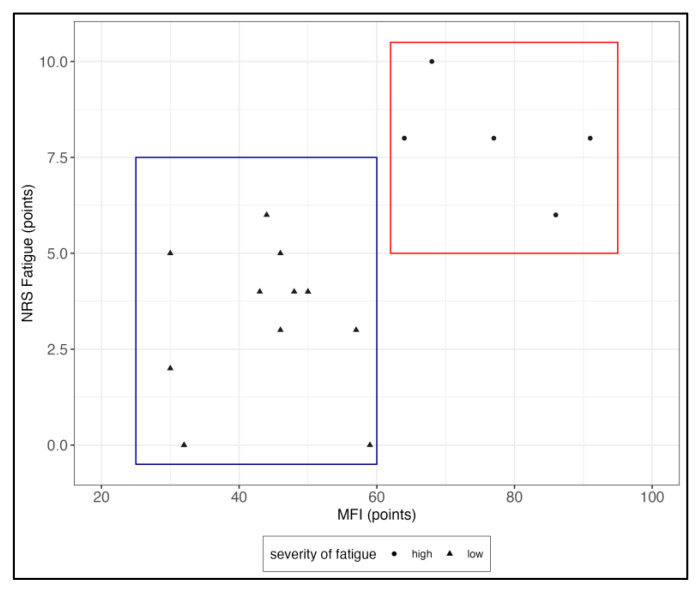
Fatigues scores in the Numeric Rating Scale (NRS) and Multidimensional Fatigue Inventory (MFI). A visual clustering of two groups is seen, dividing patients into a highly fatigued (red) and less fatigued (blue) groups.

Hierarchical Cluster analysis revealed a cluster of six patients that scored high in both the NRS fatigue and the MFI; this is visualized in Figure 1. As shown in Table 1, this group of highly fatigued pSS patients also had a higher severity of other symptoms typically associated with ME/CFS, e.g., pain, sleep disturbances and autonomic symptoms.

While scores of the HADS (*r_s_* = 0.565; *p* = 0.012), PSQI (*r_s_* = 0.678; *p* = 0.001) and NRS pain (*r_s_* = 0.460, *p* = 0.047) significantly correlated with fatigue severity in the MFI, there was no significant correlation with disease activity in the ESSDAI (*r_s_* = 0.331, *p* = 0.166), age (*r_s_* = 0.416, *p* = 0.077) or disease duration (*r_s_* = −0.143, *p* = 0.560).

**Figure A1:**
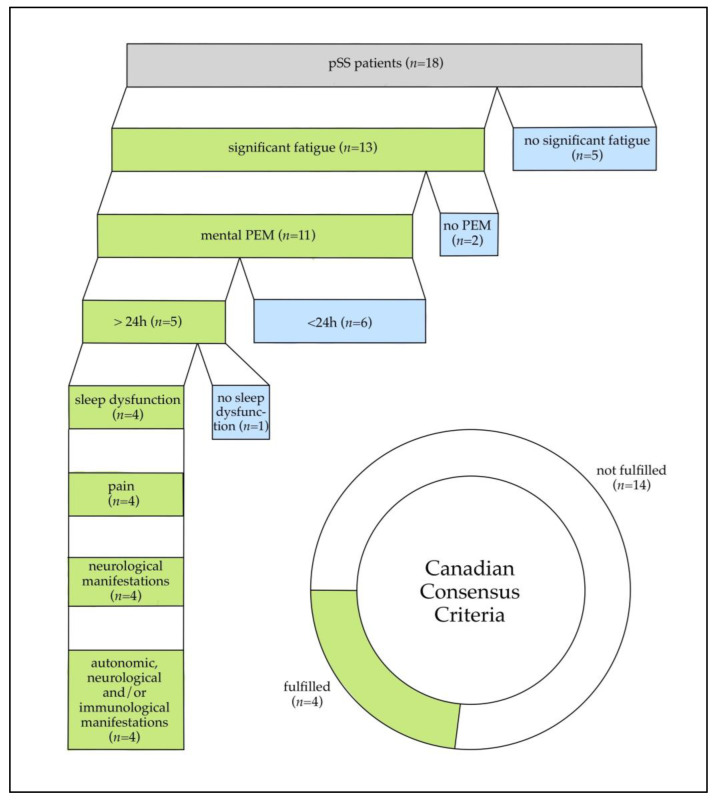
Number of patients fulfilling the Canadian Consensus Criteria (CCC) in total and per item. While patients did not describe post-exertional malaise (PEM) after physical exhaustion, 11 patients described PEM after mental and emotional exertion.

Out of 18 pSS patients that completed evaluation for the CCC (Figure A1 of the Appendix A), 13 patients fulfilled criterion 1, stating that they were experiencing significant fatigue. Eleven of those patients stated that they were experiencing PEM after mental or emotional exertion, with only five additionally agreeing that this lasted for 24 h or longer. Out of these five, four patients would have fulfilled the CCC if not for their pre-existing diagnosis of pSS. However, PEM was atypical in these four patients and only triggered by mental and emotional exertion but not physical activity.

Finally, despite scoring a median of 11 points in the ‘reduced activity’ domain of the MFI, as shown by the results of the IPAQ-SF, all but two patients managed to keep up with the WHO recommended level of physical activity. Fatigue severity in the MFI did not significantly correlate with the level of physical activity in the IPAQ-SF (*r_s_ =* −0.299; *p* = 0.288).

### 3.2. Hand Grip Strength

**Figure 2:**
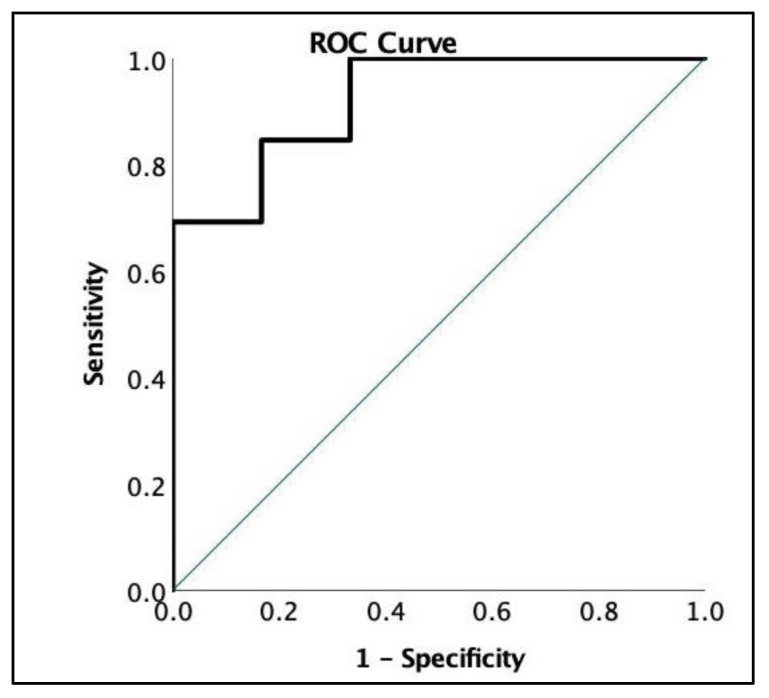
ROC analysis of Fmean1. Fmean1 reached a sensitivity of 69.2% and specificity of 100% discriminating pSS patients with high (*n* = 6) vs. low (*n* = 13) fatigue severity.

All parameters of HGS, except for the Recovery Ratio, significantly correlated with the patients′ perceived fatigue severity and, as seen in Table 1, significantly differed in patients with high compared to lower fatigue severity. ROC analysis showed a high ability to discriminate between patients with high and low perceived fatigue severity (AUC 0.923) by Fmean1 using a cutoff of <20.1 kg, proving it to be a viable method of assessing overall fatigue severity in pSS patients (Figure 2).

Adjusted for age and gender, maximum HGS (Fmax) was below the norm recommended for the general population by the dynamometer’s manufacturer in 9/19 patients. Median values are displayed in Table 1. Compared to the study by Jäkel et al. using the same HGS protocol [13], the mean HGS during the first set of measurements (Fmean1) of female pSS patients (19.28 ± 7.61 kg) was lower when compared to healthy female controls (25.6 ± 5.87 kg) but above female ME/CFS patient′s mean Fmean1 (14.6 ± 6.09 kg).

As seen in Table 1, the median Fatigue Ratio (Fmax/Fmean) was >1 in both sets of measurements, indicating a decrease in HGS during the 10 repetitions. The median Fatigue Ratio was significantly higher in the group that had high self-reported fatigue, compared to the group that reported less fatigue severity.

There was no significant difference between the initial measurements and the measurements taken after 60 min for both maximum (Fmax) (*t*(18) = 2.01; *p* = 0.06) and mean HGS (Fmean) (*t*(18) = 1.19; *p* = 0.25). Calculating the Recovery Ratio (Fmean2/Fmean1) also indicated that there was no significant decrease in HGS after the first set of measurements. A Recovery Ratio <1 indicates the inability to fully recover after 60 min, it was median 0.98 for pSS patients. Compared to female ME/CFS patients, who had a mean Recovery Ratio of 0.87 [13], this indicates that muscle strength almost fully recovers in pSS in contrast to ME/CFS.

There were no significant correlations of HGS parameters with the severity of any other symptoms including musculoskeletal pain, which might affect hand mobility. HGS parameters also did not significantly correlate with disease activity.

### 3.3. Autoantibodies

Serum levels of α1/α2-AdR-AAB, β1/β2-AdR-AAB and M3/M4-AchR-AAB were measured in 19 pSS patients. All but α2-AdR-AAB were elevated in subsets of patients compared to the autoantibody titers Loebel et al. found in healthy controls [21]. Median AAB levels were below the cutoff values, except for β2-AdR-AAB, which was elevated in the majority of pSS patients (*n* = 11/19).

**Figure 3:**
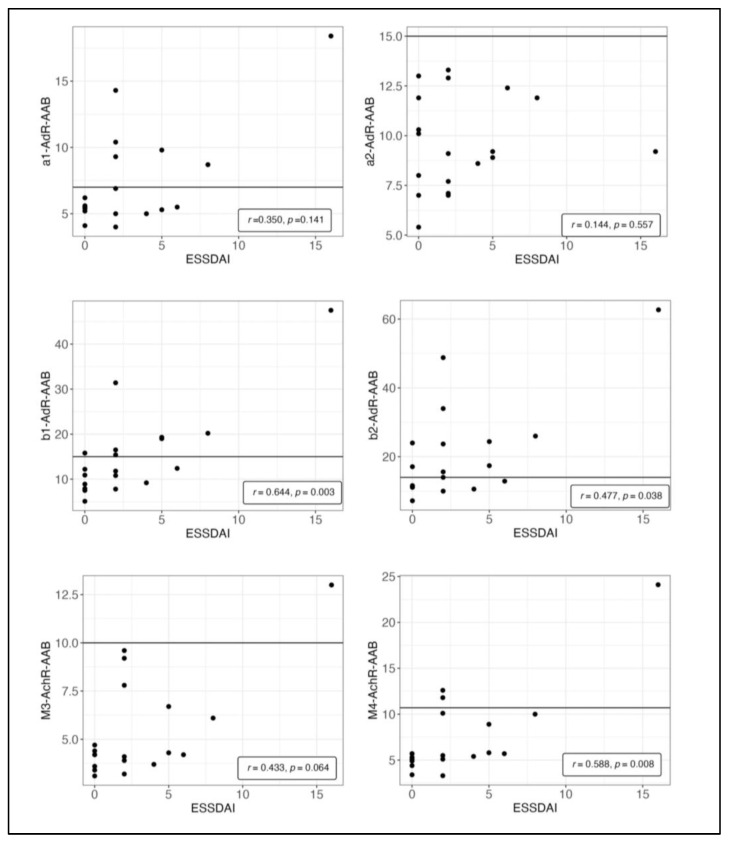
Spearman correlation between the serum levels of autoantibodies (AAB) against alpha1-, alpha2-, beta1-, beta2-adrenergic receptors (AdR) and M3- and M4-acetylcholinergic receptors (AchR) in (U/mL) and pSS disease activity (ESSDAI). Intercept lines mark cutoff values as suggested by CellTrend GmbH, Luckenwalde, Germany.

As displayed in Figure 3, levels of β1-AdR-AAB, β2-AdR-AAB and M4-AchR-AAB significantly correlated with disease activity in the total ESSDAI score, which includes 12 domains related to different organ systems. Levels of M3-AchR-AAB also correlated with the ESSDAI, but this did not reach statistical significance.

The level of α1-AdR-AAB significantly correlated with symptom severity in the ESSPRI (*r_s_* = 0.466, *p* = 0.044). Spearman correlations of AAB and any of the other clinical characteristics displayed in Table 1 did not reach statistical significance, including correlations with the scores for fatigue severity, the COMPASS 31 score and HGS measurements.

## 4. Discussion

A subgroup of ME/CFS patients is known to fulfill the pSS criteria [6,7]. Considering this finding, the hypothesis of whether there might be a significant overlap of the two diseases—clinically and pathophysiologically—was proposed. In this study, we aimed to comprehensively characterize the key clinical features of ME/CFS, fatigue, exertional intolerance and PEM symptoms among pSS patients.

### 4.1. Fatigue Severity

A cohort of 19 pSS patients of the outpatient rheumatology department was included in this study. Self-reported fatigue was evaluated by both the MFI and NRS for fatigue. Compared to healthy controls [35], pSS patients scored high in the ‘general fatigue’ domain of the MFI. This was objectifiable by HGS measurement. Firstly, measures of HGS such as Fmean and Fmax correlated highly with self-reported fatigue severity. Additionally, patients with higher fatigue severity in both the NRS and MFI had significantly higher Fatigue Ratios (Fmax/Fmean), indicating objectifiable physical fatigability within the 10 recorded repetitions.

Compared to the findings of Jäkel et al. [13], mean HGS measurements were less impaired in pSS patients compared to patients with ME/CFS, while the HGS of both populations were below the HGS of healthy controls and the expected HGS adjusted for age and sex.

Even though patients reported significant fatigue, had reduced HGS and scored high in the ‘reduced activity’ and ‘physical fatigue’ domains of the MFI, there was no significant correlation between fatigue and the reported activity level in the IPAQ-SF. Furthermore, results of the IPAQ-SF showed that almost all patients’ activity levels were in fact within a healthy range. While we did not have any comparison to the activity level these patients had before disease onset, it seems unlikely that the reduction in physical activity in pSS reaches the extent it does in patients with ME/CFS. One explanation for this lack of correlation between fatigue and physical activity is the possible absence of ‘physical exertion triggered PEM′ in pSS.

### 4.2. Exertional Intolerance and PEM

PEM is considered to be the hallmark symptom of ME/CFS and describes the worsening of symptoms after mental, emotional or physical exertion. In 129 ME/CFS patients, fatigue was the most commonly exacerbated symptom, followed by cognitive difficulties, sleep disturbances, headaches, muscle pain and flu-like feelings. Ninety percent of ME/CFS patients experienced PEM after both physical and mental exertion [40]. In contrast to this, only four pSS patients reported PEM according to the CCC definition which was triggered after mental but not physical exertion. Although a majority of pSS patients reported PEM, it only lasted a few hours in most patients.

The lack of ‘physical exertion triggered PEM’ was also in line with the normal Recovery Ratio (Fmean2/Fmean1) in pSS patients fully recovering their HGS within the 60 min time frame between measurements, with a median Recovery Ratio of 0.98. In comparison, mean HGS in healthy controls was 1.0 and 0.87 in patients with ME/CFS [13]. We interpret this nearly normal Recovery Ratio as an indication of the absence of significant fatigability after physical exertion. The finding that fatigue has not worsened after the first set of measurements further suggests a certain resilience to physical exertion.

### 4.3. Severity of Other Symptoms

Within our cohort of 19 pSS patients, we found a highly fatigued subgroup of six patients, who scored high in both the NRS fatigue and the MFI and had significantly lower HGS. This group of pSS patients presenting with severe fatigue displayed many of the symptoms associated with ME/CFS.

Firstly, this group had a higher severity of pain and sleep disturbances, both constituting key criteria of the CCC. This was to be expected, as correlations between fatigue and pain [41] as well as sleep disorders [42,43] have already been shown in different cohorts of pSS patients. Although group differences of autonomic symptom severity did not reach significance in our study, the association between fatigue and autonomic symptoms in pSS has been shown previously in other studies [29,44]. Moreover, many pSS patients fulfilled criteria from the autonomic, neurological/cognitive, neuroendocrine and immunological domains of the CCC.

Incidentally, the highly fatigued patients also had higher prevalence of anxiety and depression. This relationship has also been observed in numerous clinical studies [45,46].

Furthermore, HGS assessment proved to be a useful method for evaluating fatigue severity in patients with pSS. This cost-effective and non-invasive tool may be incorporated into the diagnostic protocol for assessing fatigue symptoms, providing a way to objectively quantify symptom load. Given the strong correlation between Fmean1 and self-reported fatigue, a reduced number of measurements may be sufficient in clinical praxis.

### 4.4. Autoantibody Correlations

Increasing evidence has suggested a potential role of neurotransmitter receptor antibodies in ME/CFS, with associations found between these antibodies and key symptoms that imply a causal pathomechanistic connection [22]. While elevations of M3-AchR-AAB in pSS are known [30,47], other neurotransmitter receptor antibodies have not been analyzed yet [48]. In our cohort of 19 pSS patients, we found elevations of α2-AdR-AAB, β1-AdR-AAB, β2-AdR-AAB, M3-AchR-AAB and M4-AchR-AAB in a subset. Remarkably, the levels of β1-AdR-AAB, β2-AdR-AAB and M4-AchR-AAB significantly correlated with systemic disease activity in the ESSDAI Score.

While the level of AAB shows correlations with clinical symptom severity in ME/CFS, including fatigue, muscle pain, cognitive impairment and autonomic dysregulation [22], in pSS, no significant correlations were observed with any of these patient reported symptoms, including fatigue or autonomic symptom severity, as well as age or disease duration.

Self-reported symptom severity assessed by ESSPRI (assessing fatigue, pain and dryness) and systemic disease activity assessed by the ESSDAI are known to show little to no correlation in pSS, indicating they should be understood as complementary [33]. The perceived severity of symptoms in pSS may be influenced by additional factors, including the patient′s comorbidities, treatment they may receive or their subjective awareness.

Given their correlation with the ESSDAI, a potential pathophysiological role of those antibodies seems conceivable and should be subject to future research. Further studies are required to investigate the potential effects of those neurotransmitter receptor autoantibodies on autonomic nervous system and vascular function in pSS, and how this may be translated into therapeutic concepts. In the case of dysfunctional AAB, therapies targeting AAB such as immunoadsorption have shown promise in subsets of ME/CFS patients [26].

### 4.5. Limitations

Limitations of our study are the lack of a head-to-head comparison of pSS with a ME/CFS patient group and the low number of pSS patients. In a follow-up study, it is important to determine whether the absence of exertion-induced post-exertional malaise (PEM) is a reliable differentiator between pSS and ME/CFS.

Furthermore, our study design did not include any healthy control subjects to compare autoantibody titers. Consequently, we rely on comparing titers to an unmatched healthy control group provided by CellTrend.

Given the disproportionately high prevalence of pSS in women compared to men, our study had limited male representation, with only one male patient included.

## 5. Conclusions

In conclusion, our study identified a subgroup of pSS patients with severe fatigue who exhibited numerous symptoms commonly associated with ME/CFS. However, only 22% of pSS patients would have fulfilled the CCC if not for their pre-existing diagnosis of pSS mostly due to the absence of PEM lasting more than 24 h. Additionally, patients with pSS reported no relevant PEM after physical exertion and showed resilience to physical exertion in repeated HGS measurements. Thus, we found the absence of ‘physical exertion triggered PEM’ to be a key difference in clinical presentation of pSS compared to ME/CFS, which may help in distinguishing between the two conditions. Furthermore, this finding is also important in disease management, as physical activity does not seem to worsen fatigue severity in pSS. Therefore, patients should not be reluctant to maintain a healthy level of physical activity.

Levels of β1-AdR-AAB, β2-AdR-AAB and M4-AchR-AAB were elevated in pSS patients and significantly correlated with the disease activity of pSS assessed by the ESSDAI. This suggests that manifestations of pSS could be mediated or aggravated by these AAB.

## Figures and Tables

**Table 1:** Clinical characteristics. Asterisks mark significant differences between groups (Mann–Whitney test, * *p* < 0.05, ** *p* < 0.01).

	Whole Cohort (*n* = 19, Median with IQR)	High Fatigue (*n* = 6, Median with IQR)	Low Fatigue (*n* = 13, Median with IQR)	High vs. Low
Sex (f/m)	18/1	6/0	12/1	*p* = 0.497
Age (years)	63 (52–68)	68.5 (52–77)	60 (54–67)	*p* = 0.253
Disease Duration (years)	7 (3–10)	7.5 (2.5–10.5)	7 (3.5–10.5)	*p* = 0.792
pSS Indices				
ESSDAI	2 (0–5)	3.5 (1.5–8.5)	2 (0–3)	*p* = 0.131
ESSPRI	4 (3.33–6)	7 (5.9–8)	4 (3.3–4.3)	*p* < 0.001 **
NRS Pain	3 (0–5)	6 (3–7.25)	3 (0–4)	*p* = 0.045 *
NRS Dryness	6 (5–9)	8.5 (5–9.25)	6 (4.5–8.5)	*p* = 0.266
NRS Fatigue	4 (3–8)	8 (7.5–8.5)	4 (1–4)	*p* < 0.001 **
MFI	50 (43–64)	72.5 (64–87.25)	46 (32–50)	*p* < 0.001 **
General Fatigue	12 (11–15)	15.5 (14.25–16.5)	12 (7.5–13.5)	*p* = 0.004 **
Physical Fatigue	10 (7–14)	15.5 (14–18.5)	8 (6.5–11)	*p* < 0.001 **
Reduced Activity	11 (7–14)	16.5 (13.5–18.5)	9 (6.5–11.5)	*p* < 0.001 **
Reduced Motivation	8 (6–13)	13 (8.75–15.5)	7 (5–8.5)	*p* = 0.005 **
Mental Fatigue	8 (6–15)	15 (11–19.25)	7 (4–12.5)	*p* = 0.010 *
HGS (kg)				
Fmean 1	20.18 (15.18–25.27)	11.73 (6.5–17.8)	24.75 (19.24–27.95)	*p* = 0.004 **
Fmean 2	19.30 (15.03–25.35)	11.36 (5.22–15.62)	23.27 (18.37–28.24)	*p* = 0.002 **
Fmax 1	25.70 (19.20–30.70)	16.55 (8.25–21.6)	27.10 (21.65–31.85)	*p* = 0.023 *
Fmax 2	21.20 (18.50–28.00)	13.8 (8.25–21.6)	25.3 (20.95–30.8)	*p* = 0.008 **
Fatigue Ratio 1	1.18 (1.09–1.36)	1.41 (1.2–1.75)	1.14 (1.09–1.22)	*p* = 0.011 *
Fatigue Ratio 2	1.11 (1.07–1.35)	1.37 (1.28–1.75)	1.08 (1.06–1.23)	*p* = 0.005 **
Recovery Ratio	0.98 (0.79–1.35)	0.85 (0.76–1.15)	0.99 (0.91–1.05)	*p* = 0.430
HADS	10 (8–15)	16.5 (11.25–24.75)	9 (5.5–11)	*p* = 0.015 *
Depression	4 (1–8)	8 (3.3–12-25)	3 (1–4.5)	*p* = 0.021 *
Anxiety	7 (5–9)	9 (6.25–14.25)	6 (3.5–7.5)	*p* = 0.084
PSQI	8 (6–13)	13.5 (9–16.25)	7 (4–11)	*p* = 0.020 *
COMPASS 31	18.22 (13.47–39.12)	43.27 (24.01–54.59)	16.46 (13.33–20.79)	*p* = 0.062
IPAQ				
MET-Minutes/Week ^(1)^	2919 (1278–6238)	1159 (0–6692)	3519 (1586–5687)	*p* = 0.223
Autoantibodies (U/mL)				
M3	4.2 (3.7–6.7)	5.45 (3.93–10.15)	4.2 (3.65–5.4)	*p* = 0.380
M4	5.7 (5.1–10)	7.3 (5.05–14.87)	5.5 (4.75–7.9)	*p* = 0.356
α1	5.5 (5.2–9.3)	7.65 (5.3–12.4)	5.5 (5.1–7.8)	*p* = 0.272
α2	9.2 (7.7–11.9)	9.65 (8.45–12.63)	9.1 (7.35–11.9)	*p* = 0.404
β1	12.2 (8.9–19)	14.45 (10.83–26.35)	10.9 (8.35–17.4)	*p* = 0.254
β2	15.6 (11.2–24.4)	20 (12.48–41.18)	14 (10.85–23.85)	*p* = 0.219
CCC (Fulfilled/Not Fulfilled)	(4/14)	(2/3)	(2/11)	*p* = 0.274

^(1)^ Only activities > 10 min in duration are taken into account. *CCC*, Canadian Consensus Criteria; *COMPASS,* Composite Autonomic Symptom Score; *ESSDAI*, the European League Against Rheumatism Sjögren′s Syndrome Disease Activity Index; *ESSPRI*, the European League Against Rheumatism Sjögren′s Syndrome Patient Reported Index; *HADS*, Hospital Anxiety and Depression Scale, *HGS*, Hand Grip Strength; *IPAQ*, International Physical Activity Questionnaire; MET, metabolic equivalent of task; *MFI,* Multidimensional Fatigue Inventory; *NRS,* Numeric Rating Scale; *pSS*, primary Sjögren′s Syndrome; *PSQI*, Pittsburgh Sleep Quality Index.

## Data Availability

The data that support the findings of this study are available from the corresponding author, L.K., upon reasonable request.

## References

[1] Qin B., Wang J., Yang Z., Yang M., Ma N., Huang F., Zhong R. (2015). Epidemiology of primary Sjogren’s syndrome: A systematic review and meta-analysis. Ann. Rheum. Dis..

[2] Brito-Zeron P., Theander E., Baldini C., Seror R., Retamozo S., Quartuccio L., Bootsma H., Bowman S.J., Dorner T., Gottenberg J.E. (2016). Early diagnosis of primary Sjogren’s syndrome: EULAR-SS task force clinical recommendations. Expert Rev. Clin. Immunol..

[3] Brito-Zeron P., Baldini C., Bootsma H., Bowman S.J., Jonsson R., Mariette X., Sivils K., Theander E., Tzioufas A., Ramos-Casals M. (2016). Sjogren syndrome. Nat. Rev. Dis. Primers.

[4] Ng W.F., Bowman S.J. (2010). Primary Sjogren’s syndrome: Too dry and too tired. Rheumatology.

[5] Miyamoto S.T., Lendrem D.W., Ng W.F., Hackett K.L., Valim V. (2019). Managing fatigue in patients with primary Sjogren’s syndrome: Challenges and solutions. Open Access Rheumatol..

[6] Nishikai M., Akiya K., Tojo T., Onoda N., Tani M., Shimizu K. (1996). ‘Seronegative’ Sjogren’s syndrome manifested as a subset of chronic fatigue syndrome. Br. J. Rheumatol..

[7] Sirois D.A., Natelson B. (2001). Clinicopathological findings consistent with primary Sjogren’s syndrome in a subset of patients diagnosed with chronic fatigue syndrome: Preliminary observations. J. Rheumatol..

[8] Sotzny F., Blanco J., Capelli E., Castro-Marrero J., Steiner S., Murovska M., Scheibenbogen C., on behalf of the European Network on ME/CFS (EUROMENE) (2018). Myalgic Encephalomyelitis/Chronic Fatigue Syndrome—Evidence for an autoimmune disease. Autoimmun. Rev..

[9] Valdez A.R., Hancock E.E., Adebayo S., Kiernicki D.J., Proskauer D., Attewell J.R., Bateman L., DeMaria A., Lapp C.W., Rowe P.C. (2018). Estimating Prevalence, Demographics, and Costs of ME/CFS Using Large Scale Medical Claims Data and Machine Learning. Front. Pediatr..

[10] Carruthers B.M., Jain A.K., De Meirleir K.L., Peterson D.L., Klimas N.G., Lerner A.M., Bested A.C., Flor-Henry P., Joshi P., Powles A.P. (2003). Myalgic Encephalomyelitis/Chronic Fatigue Syndrome. J. Chonic. Fatigue Syndr..

[11] Alunno A., Carubbi F., Bartoloni E., Cipriani P., Giacomelli R., Gerli R. (2019). The kaleidoscope of neurological manifestations in primary Sjogren’s syndrome. Clin. Exp. Rheumatol..

[12] Cafaro G., Bursi R., Chatzis L.G., Fulvio G., Ferro F., Bartoloni E., Baldini C. (2021). One year in review 2021: Sjogren’s syndrome. Clin. Exp. Rheumatol..

[13] Jakel B., Kedor C., Grabowski P., Wittke K., Thiel S., Scherbakov N., Doehner W., Scheibenbogen C., Freitag H. (2021). Hand grip strength and fatigability: Correlation with clinical parameters and diagnostic suitability in ME/CFS. J. Transl. Med..

[14] Goldblatt J., James O.F., Jones D.E. (2001). Grip strength and subjective fatigue in patients with primary biliary cirrhosis. JAMA.

[15] Strandkvist V., Andersson M., Backman H., Larsson A., Stridsman C., Lindberg A. (2020). Hand grip strength is associated with fatigue among men with COPD: Epidemiological data from northern Sweden. Physiother. Theory Prac..

[16] Chu L., Valencia I.J., Garvert D.W., Montoya J.G. (2019). Onset Patterns and Course of Myalgic Encephalomyelitis/Chronic Fatigue Syndrome. Front. Pediatr..

[17] Bartoloni E., Alunno A., Gerli R. (2019). The dark side of Sjogren’s syndrome: The possible pathogenic role of infections. Curr. Opin. Rheumatol..

[18] Stefanski A.L., Tomiak C., Pleyer U., Dietrich T., Burmester G.R., Dorner T. (2017). The Diagnosis and Treatment of Sjogren’s Syndrome. Dtsch. Arztebl. Int..

[19] Cortes Rivera M., Mastronardi C., Silva-Aldana C.T., Arcos-Burgos M., Lidbury B.A. (2019). Myalgic Encephalomyelitis/Chronic Fatigue Syndrome: A Comprehensive Review. Diagnostics.

[20] Deumer U.S., Varesi A., Floris V., Savioli G., Mantovani E., Lopez-Carrasco P., Rosati G.M., Prasad S., Ricevuti G. (2021). Myalgic Encephalomyelitis/Chronic Fatigue Syndrome (ME/CFS): An Overview. J. Clin. Med..

[21] Loebel M., Grabowski P., Heidecke H., Bauer S., Hanitsch L.G., Wittke K., Meisel C., Reinke P., Volk H.D., Fluge O. (2016). Antibodies to beta adrenergic and muscarinic cholinergic receptors in patients with Chronic Fatigue Syndrome. Brain Behav. Immun..

[22] Freitag H., Szklarski M., Lorenz S., Sotzny F., Bauer S., Philippe A., Kedor C., Grabowski P., Lange T., Riemekasten G. (2021). Autoantibodies to Vasoregulative G-Protein-Coupled Receptors Correlate with Symptom Severity, Autonomic Dysfunction and Disability in Myalgic Encephalomyelitis/Chronic Fatigue Syndrome. J. Clin. Med..

[23] Bynke A., Julin P., Gottfries C.G., Heidecke H., Scheibenbogen C., Bergquist J. (2020). Autoantibodies to beta-adrenergic and muscarinic cholinergic receptors in Myalgic Encephalomyelitis (ME) patients—A validation study in plasma and cerebrospinal fluid from two Swedish cohorts. Brain Behav. Immun. Health.

[24] Wirth K., Scheibenbogen C. (2020). A Unifying Hypothesis of the Pathophysiology of Myalgic Encephalomyelitis/Chronic Fatigue Syndrome (ME/CFS): Recognitions from the finding of autoantibodies against ss2-adrenergic receptors. Autoimmun. Rev..

[25] Fujii H., Sato W., Kimura Y., Matsuda H., Ota M., Maikusa N., Suzuki F., Amano K., Shin I., Yamamura T. (2020). Altered Structural Brain Networks Related to Adrenergic/Muscarinic Receptor Autoantibodies in Chronic Fatigue Syndrome. J. Neuroimaging.

[26] Tolle M., Freitag H., Antelmann M., Hartwig J., Schuchardt M., van der Giet M., Eckardt K.U., Grabowski P., Scheibenbogen C. (2020). Myalgic Encephalomyelitis/Chronic Fatigue Syndrome: Efficacy of Repeat Immunoadsorption. J. Clin. Med..

[27] Scheibenbogen C., Loebel M., Freitag H., Krueger A., Bauer S., Antelmann M., Doehner W., Scherbakov N., Heidecke H., Reinke P. (2018). Immunoadsorption to remove ss2 adrenergic receptor antibodies in Chronic Fatigue Syndrome CFS/ME. PLoS ONE.

[28] Davies K., Ng W.F. (2021). Autonomic Nervous System Dysfunction in Primary Sjogren’s Syndrome. Front. Immunol..

[29] Newton J.L., Frith J., Powell D., Hackett K., Wilton K., Bowman S., Price E., Pease C., Andrews J., Emery P. (2012). Autonomic symptoms are common and are associated with overall symptom burden and disease activity in primary Sjogren’s syndrome. Ann. Rheum. Dis..

[30] Fayyaz A., Kurien B.T., Scofield R.H. (2016). Autoantibodies in Sjogren’s Syndrome. Rheum. Dis. Clin. N. Am..

[31] Shiboski C.H., Shiboski S.C., Seror R., Criswell L.A., Labetoulle M., Lietman T.M., Rasmussen A., Scofield H., Vitali C., Bowman S.J. (2017). 2016 American College of Rheumatology/European League Against Rheumatism classification criteria for primary Sjogren’s syndrome: A consensus and data-driven methodology involving three international patient cohorts. Ann. Rheum. Dis..

[32] Dachverband Osteologie e.V (2017). Prophylaxe, Diagnostik und Therapie der Osteoporose bei Postmenopausalen Frauen und Männern—Leitlinie des Dachverbands der Deutschsprachigen Wissenschaftlichen Osteologischen Gesellschaften e.V..

[33] Seror R., Theander E., Brun J.G., Ramos-Casals M., Valim V., Dorner T., Bootsma H., Tzioufas A., Solans-Laque R., Mandl T. (2015). Validation of EULAR primary Sjogren’s syndrome disease activity (ESSDAI) and patient indexes (ESSPRI). Ann. Rheum. Dis..

[34] Smets E.M., Garssen B., Bonke B., De Haes J.C. (1995). The Multidimensional Fatigue Inventory (MFI) psychometric qualities of an instrument to assess fatigue. J. Psychosom. Res..

[35] Hewlett S., Dures E., Almeida C. (2011). Measures of fatigue: Bristol Rheumatoid Arthritis Fatigue Multi-Dimensional Questionnaire (BRAF MDQ), Bristol Rheumatoid Arthritis Fatigue Numerical Rating Scales (BRAF NRS) for severity, effect, and coping, Chalder Fatigue Questionnaire (CFQ), Checklist Individual Strength (CIS20R and CIS8R), Fatigue Severity Scale (FSS), Functional Assessment Chronic Illness Therapy (Fatigue) (FACIT-F), Multi-Dimensional Assessment of Fatigue (MAF), Multi-Dimensional Fatigue Inventory (MFI), Pediatric Quality Of Life (PedsQL) Multi-Dimensional Fatigue Scale, Profile of Fatigue (ProF), Short Form 36 Vitality Subscale (SF-36 VT), and Visual Analog Scales (VAS). Arthritis Care Res..

[36] Bjelland I., Dahl A.A., Haug T.T., Neckelmann D. (2002). The validity of the Hospital Anxiety and Depression Scale. An updated literature review. J. Psychosom. Res..

[37] Buysse D.J., Reynolds C.F., Monk T.H., Berman S.R., Kupfer D.J. (1989). The Pittsburgh Sleep Quality Index: A new instrument for psychiatric practice and research. Psychiatry Res..

[38] Craig C.L., Marshall A.L., Sjostrom M., Bauman A.E., Booth M.L., Ainsworth B.E., Pratt M., Ekelund U., Yngve A., Sallis J.F. (2003). International physical activity questionnaire: 12-country reliability and validity. Med. Sci. Sports Exerc..

[39] Sletten D.M., Suarez G.A., Low P.A., Mandrekar J., Singer W. (2012). COMPASS 31: A refined and abbreviated Composite Autonomic Symptom Score. Mayo Clin. Proc..

[40] Chu L., Valencia I.J., Garvert D.W., Montoya J.G. (2018). Deconstructing post-exertional malaise in myalgic encephalomyelitis/ chronic fatigue syndrome: A patient-centered, cross-sectional survey. PLoS ONE.

[41] Koh J.H., Kwok S.K., Lee J., Son C.N., Kim J.M., Kim H.O., Park S.H., Sung Y.K., Choe J.Y., Lee S.S. (2017). Pain, xerostomia, and younger age are major determinants of fatigue in Korean patients with primary Sjogren’s syndrome: A cohort study. Scand J. Rheumatol..

[42] Hsieh M.C., Hsu C.W., Lu M.C., Koo M. (2019). Increased risks of psychiatric disorders in patients with primary Sjogren’s syndrome-a secondary cohort analysis of nationwide, population-based health claim data. Clin. Rheumatol..

[43] Priori R., Minniti A., Antonazzo B., Fusconi M., Valesini G., Curcio G. (2016). Sleep quality in patients with primary Sjogren’s syndrome. Clin. Exp. Rheumatol..

[44] Cai F.Z., Lester S., Lu T., Keen H., Boundy K., Proudman S.M., Tonkin A., Rischmueller M. (2008). Mild autonomic dysfunction in primary Sjogren’s syndrome: A controlled study. Arthritis Res. Ther..

[45] Cui Y., Xia L., Li L., Zhao Q., Chen S., Gu Z. (2018). Anxiety and depression in primary Sjogren’s syndrome: A cross-sectional study. BMC Psychiatry.

[46] Segal B., Thomas W., Rogers T., Leon J.M., Hughes P., Patel D., Patel K., Novitzke J., Rohrer M., Gopalakrishnan R. (2008). Prevalence, severity, and predictors of fatigue in subjects with primary Sjogren’s syndrome. Arthritis Rheum..

[47] Bacman S., Sterin-Borda L., Camusso J.J., Arana R., Hubscher O., Borda E. (1996). Circulating antibodies against rat parotid gland M3 muscarinic receptors in primary Sjogren’s syndrome. Clin. Exp. Immunol..

[48] Nardi N., Brito-Zeron P., Ramos-Casals M., Aguilo S., Cervera R., Ingelmo M., Font J. (2006). Circulating auto-antibodies against nuclear and non-nuclear antigens in primary Sjogren’s syndrome: Prevalence and clinical significance in 335 patients. Clin. Rheumatol..

